# Squalene production under oxygen limitation by *Schizochytrium* sp. S31 in different cultivation systems

**DOI:** 10.1007/s00253-024-13051-3

**Published:** 2024-02-13

**Authors:** Lina Schütte, Patrick G. Hanisch, Nina Scheler, Katharina C. Haböck, Robert Huber, Franziska Ersoy, Ralf G. Berger

**Affiliations:** 1 Institute of Food Chemistry, Gottfried Wilhelm Leibniz University Hannover, Hannover, Germany; 2 Department of Engineering and Management, Munich University of Applied Sciences HM, Munich, Germany

**Keywords:** Squalene, *Schizochytrium* sp. S31, Sterols, RQ, Backscatter, Continuous cultivation, Thraustochytrids

## Abstract

**Abstract:**

The triterpene squalene is widely used in the food, cosmetics and pharmaceutical industries due to its antioxidant, antistatic and anti-carcinogenic properties. It is usually obtained from the liver of deep sea sharks, which are facing extinction. Alternative production organisms are marine protists from the family *Thraustochytriaceae*, which produce and store large quantities of various lipids. Squalene accumulation in thraustochytrids is complex, as it is an intermediate in sterol biosynthesis. Its conversion to squalene 2,3-epoxide is the first step in sterol synthesis and is heavily oxygen dependent. Hence, the oxygen supply during cultivation was investigated in our study. In shake flask cultivations, a reduced oxygen supply led to increased squalene and decreased sterol contents and yields. Oxygen-limited conditions were applied to bioreactor scale, where squalene accumulation and growth of *Schizochytrium* sp. S31 was determined in batch, fed-batch and continuous cultivation. The highest dry matter (32.03 g/L) was obtained during fed-batch cultivation, whereas batch cultivation yielded the highest biomass productivity (0.2 g/L*h^−1^). Squalene accumulation benefited from keeping the microorganisms in the growth phase. Therefore, the highest squalene content of 39.67 ± 1.34 mg/g was achieved by continuous cultivation (D = 0.025 h^−1^) and the highest squalene yield of 1131 mg/L during fed-batch cultivation. Volumetric and specific squalene productivity both reached maxima in the continuous cultivation at D = 0.025 h^−1^ (6.94 ± 0.27 mg/L*h^−1^ and 1.00 ± 0.03 mg/g*h^−1^, respectively). Thus, the choice of a suitable cultivation method under oxygen-limiting conditions depends heavily on the process requirements.

**Key points:**

• *Measurements of respiratory activity and backscatter light of thraustochytrids*

• *Oxygen limitation increased squalene accumulation in Schizochytrium sp. S31*

• *Comparison of different cultivation methods under oxygen-limiting conditions*

**Supplementary information:**

The online version contains supplementary material available at 10.1007/s00253-024-13051-3.

## Introduction

Squalene is a polyunsaturated, linear triterpene (C_30_H_50_) consisting of six isoprene units. It was first found in the liver oil of deep sea sharks and named after the shark family *Squalidae* (Tsujimoto 1916). The compound naturally occurs in higher organisms and plays an important role as an intermediate in sterol synthesis (Nes 2011; Xu et al. 2004). Squalene is also ubiquitously present in human tissue, especially in the skin sebum, which contains up to 12% of the triterpene, and where it is presumed to protect against oxidative stress such as UV light (Nicolaides 1974; Passi et al. 2002). Due to its antioxidative and hydrating properties, it is often used in cosmetics. Furthermore, squalene is of interest for pharmaceutical applications because it was reported to have antitumor, anti-inflammatory and cardioprotective effects and can be used as an adjuvant or drug carrier (Reddy and Couvreur 2009; Spanova and Daum 2011).

Due to its high abundance in deep sea sharks, squalene is traditionally obtained from their liver oil, which causes overfishing of these endangered species as well as environmental problems. Another source of squalene, albeit in much lower concentration, are plants such as olives, amaranth, wheat and rice (Yarkent and Oncel 2022). Their cultivation for squalene production suffers from high demands for arable land and potential competition with food production. Therefore, alternative sustainable sources are of great interest (Wang et al. 2015).

Thraustochytrids are heterotrophic eukaryotes from the family *Thraustochytriaceae*, which belongs to the class *Labyrinthulea*. These unicellular protists commonly occur in nutrient-rich seawater and sediments found in mangrove forests (Aasen et al. 2016; Fossier Marchan et al. 2018). Depending on their cultivation conditions, they accumulate up to 50% lipids in their dry matter, which makes them potential candidates for microbial terpene production (Morabito et al. 2019). In thraustochytrids, squalene is synthesised via the mevalonate pathway, starting with acetyl-CoA and yielding farnesyl diphosphate (FPP), which is subsequently converted to squalene (Nes 2011; Spanova and Daum 2011). The first step in sterol synthesis is the oxygenation of squalene to squalene 2,3-epoxide catalysed by the squalene monooxygenase (Jiang et al. 2020; Ono 2002). This oxygen-dependent reaction heavily influences the accumulation of squalene (Fan et al. 2010). Oxygen-limited conditions have been used to limit the conversion of squalene to squalene 2,3-epoxide and thereby boost squalene yield. Low oxygen supply had a positive effect on squalene content and a negative effect on the biosynthesis of sterols in the thraustochytrid strain ACEM 6063 (Lewis et al. 2001).

For industrial squalene production, a transfer to the bioreactor is necessary. *Schizochytrium* sp. S31 has been cultivated in bioreactors under oxygen-limited conditions for docosahexaenoic acid (DHA) production (Chang et al. 2014; Guo et al. 2016). For the same product, continuous cultivation of thraustochytrids has been described (Ethier et al. 2011; Ganuza and Izquierdo 2007; Pawar et al. 2021). The differences between batch and fed-batch cultivation regarding squalene formation were investigated by Ha et al. (2017) and Hoang et al. (2018) without oxygen limitation. However, a comparative study of squalene production in batch, fed-batch and continuous cultivations under oxygen limitation is missing. Additionally, online monitoring of respiratory activity and growth *via* backscatter in shake flasks have not been performed with thraustochytrids yet.

The aim of this work was the detailed investigation of the impact of oxygen-limiting conditions on squalene formation and growth of the thraustochytrid *Schizochytrium* sp. S31. For this, real-time monitoring of respiratory data and backscatter were performed in shake flasks. In addition, the findings were transferred to bioreactor scale for comparison of the most common cultivation systems (batch, fed-batch and continuous).

## Materials and methods

### Reagents

All chemicals were purchased from Carl Roth (Karlsruhe, Germany), Sigma Aldrich (Darmstadt, Germany), Merck Millipore (Darmstadt, Germany), Riedel-de Haën AG (Seelze, Germany), Honeywell (Charlotte, USA), VWR (Darmstadt, Germany) and Thermo Fisher Scientific (Waltham, USA) if not stated otherwise. All organic solvents were bought from Carl Roth (Karlsruhe, Germany) and Supelco (Bellefront, USA). All solvents were bought in analytical grade for gas chromatography (methanol and chloroform) or purified to analytical grade by in-house distillation (hexane).

### Cultivation of *Schizochytrium* sp. S31

#### Cultivation in shake flasks

*Schizochytrium* sp. S31 (American Type culture collection (ATCC) 20888) was preserved in 25% (v/v) glycerol with preculture medium (PM, 10 g d-glucose, 1 g yeast extract, 1 g peptone ex casein, 12.5 g NaCl, 0.5 g KCl, 2.5 g MgSO4 · 7 H_2_O, 1 g monosodium glutamate, 6 mg ammonium iron (III) citrate, 51.5 mg CaCl_2_, 30.4 mg K_2_HPO_4_, 2.86 mg H_3_BO_3_, 1.81 mg MnCl_2_ · 4 H_2_O, 0.222 mg ZnSO_4_ · 7 H_2_O, 0.39 mg Na_2_MoO_4_ · 2 H_2_O, 0.079 mg CuSO_4_ · 2 H_2_O, 0.0477 mg CoSO_4_ · 7 H_2_O or 0.0494 mg Co(NO_3_)_2_ · 6 H_2_O, 0.1 mg carbenicillin, 595.8 mg HEPES, 0.5 µg biotin, 100 µg thiamine hydrochloride and 0.5 µg cobalamin per litre) at − 80 °C. For precultures, 750 µL glycerol stock was transferred to 75 mL preculture medium (PM w/o agar) in a 250-mL shake flask and incubated at 120 rpm, 28 °C for 48 h (shaking diameter 50 mm). Standard medium (SM) was used for main shake flask cultivations (PM, but with 50 g d-glucose, 2.78 g yeast extract, 2.78 g peptone ex casein per litre). A KuhnerTOM (Kuhner Shaker GmbH, Germany) and a backscatter measurement system (Cell Growth Quantifier (CGQ), SPI Europe, Germany) were used to determine respiratory data (OTR: oxygen transfer rate, CTR: carbon dioxide evolution rate, RQ: respiratory quotient) and growth for two shake flasks of each measurement series. The KuhnerTOM system was set to an airflow of 20 mL/min during the aeration phase. All shake flasks were incubated at 120 rpm, 28 °C and a shaking diameter of 50 mm. For each sampling point, a whole shake flask was harvested (three during the growth phase and two during the stationary phase).

#### Bioreactor cultivation

50 mL PM w/o agar was inoculated with cells from an agar plate (1.5% agar w/v) and incubated for two days at 28 °C. Then, 75 mL PM w/o agar was inoculated with 5% (*v/v*) of the first preculture and incubated as described before. After 48 h, the second preculture was used to inoculate the bioreactor (5% *v/v*).

Batch, fed-batch and continuous cultivations of *Schizochytrium* sp. S31 were carried out in a 6-L stirred tank reactor (Minifors 2, Infors AG, Switzerland) with a total working volume of 3 L. SM was used for bioreactor cultivations. All bioreactor cultivations ran at 28 °C with a stirring speed of 350 rpm and a constant airflow of 2 L/min. 3 mL rapeseed oil was added at the beginning of the fermentation to avoid excessive foaming. The bioreactor was equipped with two Rushton turbines with a distance of 7.5 cm. For DO and pH measurements, a Visiferm DO sensor and an Easyferm 325 pH electrode (Hamilton Bonaduz AG, Switzerland) were used. A BlueInOne Ferm gas analyser (BlueSens GmbH, Germany) was connected to the bioreactor’s exhaust gas flow to determine its composition. Respiratory data (CER, OUR, RQ) were calculated from the exhaust gas composition using a softsensor implemented in the bioreactor’s software (eve®, Infors AG, Switzerland):

1 $$OUR=\left({C}_{{O}_{2}}^{{\text{in}}}-{C}_{{O}_{2}}^{{\text{out}}}\right)\bullet \frac{Q}{V}$$

2 $$CER=\left({C}_{{Co}_{2}}^{{\text{out}}}-{C}_{{CO}_{2}}^{{\text{in}}}\right)\bullet \frac{Q}{V}$$

3 $$RQ=\frac{CER}{OUR}$$

where *Q* is the airflow, *V* is the culture volume, ${C}_{{O}_{2}}^{{\text{in}}}$ and ${C}_{{CO}_{2}}^{{\text{in}}}$ are the concentration of O_2_ and CO_2_ in the air and ${C}_{{O}_{2}}^{{\text{out}}}$ and ${C}_{{Co}_{2}}^{{\text{out}}}$ in the exhaust gas.

During batch and fed-batch fermentations, samples were taken every 24 h. Feeding of yeast extract and d-glucose for fed-batch cultivations started after 48 h. Initially, 18 g/L d-glucose and 1 g/L yeast extract were fed discontinuously every 24 h. After 96 h, the d-glucose feed was adjusted to 10 g/L every 24 h.

Continuous cultivation was started after 48 h of initial batch operation. SM was used as a feed medium. A peristaltic pump with four channels (Ismatec IPC 4, Cole-Parmer Instrument Company, USA) was used for the simultaneous feeding of the cultivation medium and harvesting of fermentation broth. After the start of the continuous operation, samples for dry matter determination were taken two times a day (after 8 h and 16 h). Steady state was considered as reached after at least three volume changes of fermentation medium and fluctuations in dry matter less than 5%. Three samples of each dilution rate, which met these requirements, were used for offline analyses.

### Analytical methods

#### Determination of dry matter

Samples were centrifuged at 4676 rcf (relative centrifugal force) for 10 min. 1 mL of supernatant was stored at − 20 °C for d-glucose quantification. The pellet was washed once with an amount of demineralized water equal to the sample volume. Swimming cells were harvested and washed by vacuum filtration (cellulose-acetate filter, 0.45 µm). Cell pellets and/or filters were freeze-dried for at least 24 h (Alpha 1–4 LSCbasic, Martin Christ Gefriertrocknungsanlagen GmbH, Germany). Dry matter was determined gravimetrically.

#### d-glucose quantification

d-glucose in the fermentation supernatant was measured using a d-glucose assay kit (GOPOD format, Megazyme, Ireland) according to the manufacturer’s protocol. External calibration with d-glucose standards was performed.

#### Squalene and sterol extraction

Squalene and sterol extraction from the freeze-dried cells were performed in a two-step process: First, the total lipid content was extracted, followed by purification of the unsaponifiable matter. Extraction of total lipid content was performed as described by Bligh and Dyer (1959). Methanol, chloroform and 0.8% KCl were added to the freeze-dried cells in a ratio of 1:2:0.8 (v/v/v). After rigorous mixing for 30 min, chloroform and 0.8% KCl were added to obtain a final methanol-chloroform-0.8% KCl ratio of 2:2:1.8 (v/v/v). Phase separation was achieved by centrifugation (4676 × g, 20 min). The lower phase was transferred to a new vial, and the chloroform was removed by evaporation under nitrogen. For the separation of unsaponifiable matter (containing squalene and sterols), 15% potassium hydroxide (w/v) in methanol-water (4:1, v/v) was added to the samples. Saponification of the samples was carried out at 60 °C for 3 h. Afterwards, the unsaponifiable matter was extracted three times with hexane, and the extract was stored at − 20 °C until analysis. Phase separation was achieved each time by centrifugation (4676 rcf, 10 min). Octadecylbenzene (ODB) was used as an internal standard to monitor recovery.

#### Squalene and sterol identification and quantification

Squalene and sterol separation and identification were achieved by gas chromatography-mass spectrometry. GC–MS analysis was performed on an Agilent GC-7890B coupled to a 5977A mass selective detector (Agilent Technologies, USA). The instrument was equipped with a DB-5MS UI fused-silica capillary column (30 m × 0.25 mm, 0.25 µm film thickness). The temperatures of the transfer line, the ion source and the quadrupole were set to 330 °C, 230 °C and 150 °C, respectively. Electron impact ionisation was performed at 70 mV, and the scan range was set to 33–600 amu (*m/z*). Analyses of the 0.5 µL sample were performed with a carrier gas (He) flow of 1 mL/min. The temperature gradient used for separation started at 40 °C (holding for 3 min) and increased by 10 °C/min until 325 °C (holding for 10 min). Structural determination of squalene and sterols was carried out by comparison with reference spectra from the NIST Standard Reference Database. Alkanes from C_21_ to C_40_ were used for the calculations of retention indices.

Squalene and sterol separation and quantification were achieved by gas chromatography. For GC measurements, a Shimadzu GC-2010 gas chromatograph equipped with a DB-5MS UI fused-silica capillary column (30 m × 0.32 mm, 0.25 µm film thickness), an AOC-20 s autosampler, a split/splitless injector and a flame ionization detector were used. Analyses were performed with a carrier gas (H_2_) flow of 5.18 mL/min and a split of 1:5. Injector and detector temperatures were set to 350 °C. Prior to analysis, the samples were filtered through a 0.45-µm polytetrafluoroethylene (PTFE) membrane. Separation of 1 µL sample was performed by applying a temperature gradient starting at 130 °C (holding for 3 min) and increasing by 10 °C/min until 325 °C (holding for 10 min). Alkanes from C_21_ to C_40_ were used for the calculation of retention indices. Squalene and sterol quantification were performed by external calibration with analytical standards (squalene and cholesterol).

### Data analysis

The software OriginPro was used to determine the correlation of backscatter signal and dry matter at high cell densities. The linear fit was performed using the York method considering *y*- and *x*-errors.

## Results

### Effect of oxygen supply on dry matter formation in shake flask cultivations

**Fig. 1:**
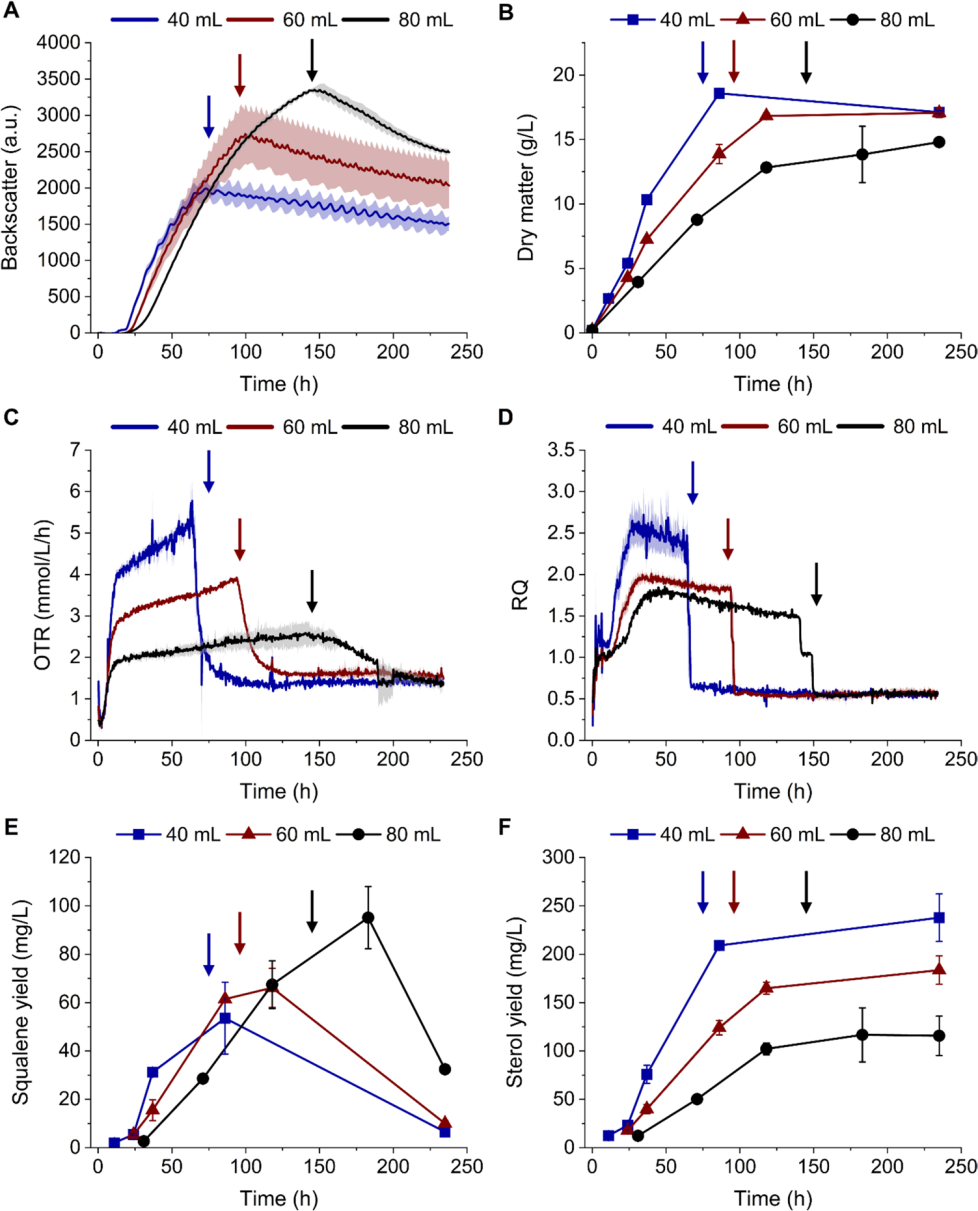
Effect of the oxygen transfer rate on biomass formation and squalene/sterol accumulation in *Schizochytrium* sp. S31 in shake flasks with varying filling volumes (blue: 40 mL; red: 60 mL; black: 80 mL). Arrows indicate the start of the stationary phase for each filling volume based on the backscatter signal. (**A**) Backscatter light, (**B**) dry matter, (**C**) oxygen transfer rate, (**D**) respiratory quotient, (**E**) squalene and (**F**) sterol yield per litre cultivation volume over cultivation time**.** Data presented are the mean of duplicates ± standard deviation

**Fig. 2:**
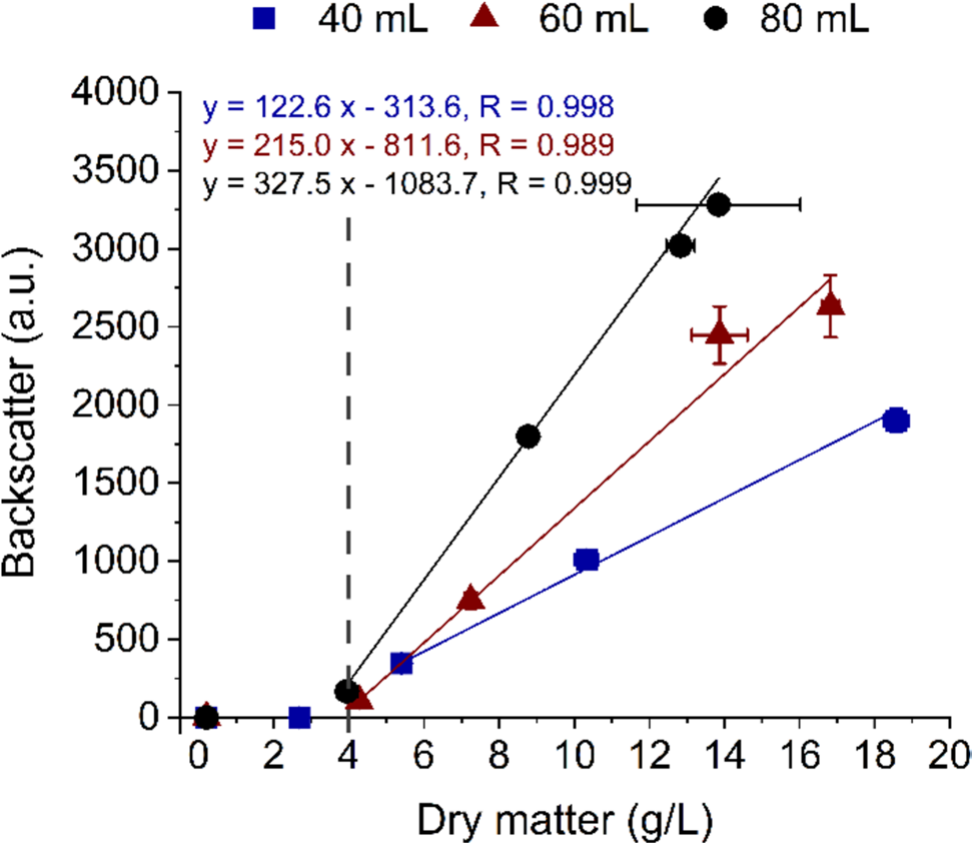
Correlation of backscatter light and dry matter during cultivations of *Schizochytrium* sp. S31. Cultivations were performed in shake flasks with varying filling volumes (blue squares, 40 mL; red triangles, 60 mL; black circles, 80 mL). Starting from 4 g/L, regression lines and the corresponding equations of the linear regressions and Pearson correlation coefficients are shown

Growth monitoring of *Schizochytrium* sp. S31 in shake flask cultivations was performed by backscatter light measurements (CGQ), a quick and non-invasive alternative for growth monitoring of cluster-forming microorganisms, and gravimetric determination of dry matter. CGQ-assisted shake flask cultivations were performed using varying filling volumes (40 mL, 60 mL and 80 mL) to generate different oxygen supplies as verified by the OTR measured via KuhnerTOM-system (Fig. 1A–D). The fastest growth and the highest biomass formation (18.6 ± 0.33 g/L) were achieved with the lowest filling volume (40 mL) and thus the highest oxygen supply. The consumption rate of the main carbon source glucose increased with oxygen supply (Supplementary Fig. S1A). In general, the backscatter signal increased with the shake flask filling volume, which is typical for measurements performed through the bottom of the shake flask. According to the backscatter measurements, the thraustochytrid reached the stationary phase after 75 h, 96 h and 145 h (for 40 mL, 60 mL and 80 mL, respectively). These time points coincide with the time of glucose depletion (Supplementary Fig. S1A). The exact start of the stationary phase is not visible in the dry matter plot due to a lower data density, but the trend fits the backscatter measurements. The backscatter signal and dry matter displayed a linear correlation at higher cell densities (> 4 g/L) (Fig. 2). The last datapoint for each data set was excluded from the graph because the backscatter signal decreased slowly after the stationary phase was reached.

### Effect of oxygen supply on squalene accumulation in shake flask cultivations

All cultivations were oxygen limited as indicated by the restricted OTR courses during the growth phase (Fig. 1C). In contrast to the slightly rising OTR curve of the 40-mL and 60-mL cultivations between 14 h and their respective start of the stationary phase, a plateau in the OTR curve of the 80-mL cultivation is clearly visible. The maximum OTR (5.78 mmol/L*h^−1^, 3.93 mmol/L*h^−1^ and 2.60 mmol/L*h^−1^ for 40 mL, 60 mL and 80 mL filling volume, respectively) were determined just before the OTR declined rapidly. This drop in oxygen transfer indicated the beginning of the stationary phase, which is illustrated by the corresponding arrows. The maximum RQ values (Fig. 1D) decreased with increasing filling volume (approximately 2.6, 2.0 and 1.8 for 40 mL, 60 mL and 80 mL, respectively).

To determine the effect of oxygen supply on squalene accumulation over the course of the cultivation, three samples were taken during the growth and two during the stationary phase. Supplementary Fig. S1B demonstrates that squalene accumulated during cell growth and started to decrease in the stationary phase. Additionally, it decreased with oxygen supply. The squalene yield (Fig. 1E) decreased analogously and was maximal in the early stationary phase (53.57 mg/L, 66.13 mg/L and 95.11 mg/L for 40 mL, 60 mL and 80 mL, respectively). In general, sterol content and yield increased over the course of the cultivation and stabilised in the stationary phase (Fig. 1F and Supplementary Fig. S1C). Both sterol content and yield increased with oxygen supply. The maximum sterol yield was achieved in the stationary phase (237.80 mg/L, 183.57 mg/L and 116.67 mg/L for 40 mL, 60 mL and 80 mL, respectively).

### Transfer of oxygen-limiting conditions to bioreactor scale

**Fig. 3:**
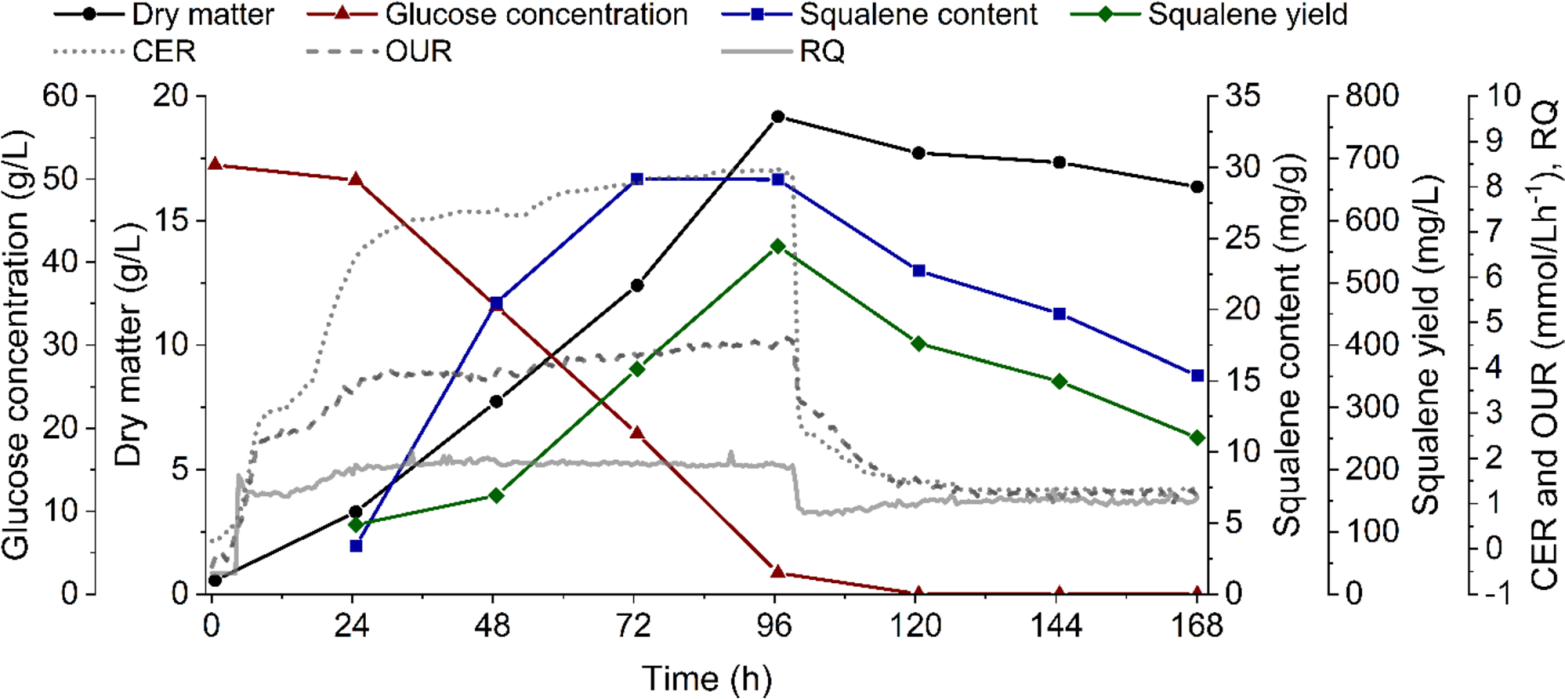
Batch cultivation of *Schizochytrium* sp. S31 in a stirred tank reactor under oxygen-limited conditions. Depicted are the dry matter (black circles), glucose concentration (red triangles), squalene content (blue squares), squalene yield (green diamonds), carbon dioxide evolution rate (CER, dotted line), oxygen uptake rate (OUR, dashed line) and respiratory quotient (RQ, solid line) over cultivation time

Oxygen-limited conditions from shake flasks were transferred to different cultivation strategies in a 6-L stirred tank reactor (3 L working volume). The shake flask cultivations (Fig. 1) with 60 mL filling volume most closely resembled the bioreactor batch cultivation (Fig. 3) regarding oxygen input: maximum OTR/OUR (approximately 4 mmol/L/h and 4.5 mmol/L/h for shake flasks and bioreactor, respectively) and RQ values (approximately 1.9 and 2 for shake flasks and bioreactor, respectively) for both cultivation systems were highly similar. Similar to the shake flask cultivations, slightly rising OUR and CER courses were attributed to the release of metabolites or the decrease in fermentation volume over time. The stationary phase of the 60-mL shake flask cultivations started close to that of the bioreactor batch cultivation (96 h and 100 h, respectively). In the bioreactor, significantly higher values for dry matter (19.18 g/L), squalene content (29.14 mg/g) and yield (559 mg/L) were achieved. As in all shake flasks, maximal values were reached just before the beginning of the stationary phase (96 h), which was indicated by a drop in respiratory data. Squalene content and yield dropped after 96 h and continued to fall during the progression of the stationary phase.

The elongation of an organism’s growth phase is known to be beneficial for the production of growth-coupled products like squalene (Yang et al. 2022). Therefore, fed-batch cultivation was performed in the bioreactor under the same conditions as the batch experiment. Starting after 48 h, 1 g/L yeast extract and initially 18, after 96 h, 10 g/L glucose were fed every 24 h. Squalene content benefited from high glucose levels in previous experiments (data not shown). Therefore, glucose levels were kept above 25 g/L during fed-batch cultivation in this study. Time points of the feed were accompanied by drops in respiratory data. Compared to the batch cultivation, higher values for dry matter (31.03 g/L), squalene content (38.35 mg/g) and yield (1131 mg/L) were achieved. Maximal values were reached just before the culture started to suffer from an unknown substrate limitation (216 h), as indicated by a final drop in respiratory data and the accumulation of glucose.

To elongate the growth phase and thus elevate squalene levels even further, continuous cultivation was established. Cultivation parameters were identical to the two previous cultivations. Dilution rates were adjusted to 0.015 h^−1^, 0.02 h^−1^, 0.025 h^−1^ and 0.03 h^−1^. For each dilution rate, three samples were taken after a steady state was reached (Fig. 5). In general, glucose concentrations rose with the dilution rate (22.63 to 40.52 g/L). Dry matter increased from D = 0.015 to 0.02 h^−1^ (max. 8.30 ± 0.23 g/L) and decreased with further increasing dilution rates (Fig. 5A). Biomass productivity was likewise maximal at a dilution rate of 0.02 h^−1^ (0.177 ± 0.003 g/L*h^−1^) (Fig. 5B). The biomass yield coefficient Y_X/S_ increased with the dilution rate (max. 0.03 h^−1^: 0.56 ± 0.07 g/g). Regarding squalene production, the yield per litre peaked for D = 0.02 h^−1^ with 293 ± 6 mg/L, while the yield per dry matter was maximal for 0.025 h^−1^ with 39.67 ± 1.33 mg/g. The latter corresponded to the overall optimum for squalene productivity as well as the squalene yield coefficient (6.94 ± 0.27 mg/L*h^−1^ and 39.67 ± 1.34 mg/g, respectively) (Fig. 5C, D).

### Comparison of different cultivation systems under oxygen-limiting conditions

A comparison of the biomass formation and squalene production obtained by different cultivation methods is given in Table 1. The dry matter of the fed-batch cultivation was about double that of the batch and 4.5 × higher than that of the continuous cultivation. Biomass productivity, on the other hand, benefited most from batch cultivation. The squalene content achieved by continuous cultivation (D = 0.025 h^−1^) and fed-batch cultivation was about 30% higher compared to batch cultivation. Regarding squalene yield, the fed-batch cultivation outperformed the batch cultivation by a factor of two and the continuous cultivation by a factor of four. Due to the oxygen limitation, maximum specific growth rates were limited and comparable in batch and fed-batch cultivations. The specific growth rate of the continuous cultivation was determined by the dilution rate. The squalene productivities of the continuous cultivation (D = 0.025 h^−1^) surpassed those of the batch (by 17 and 70% for volumetric and specific squalene productivity, respectively) and fed-batch cultivations (by 25 and 80% for volumetric and specific squalene productivity, respectively). The biomass and squalene yield/biomass coefficients Y_P/S_, Y_X/S_ and Y_P/X_ benefited the most from continuous cultivation. Compared to the batch cultivation, they increased by 41%, 19% and 24% and compared to the fed-batch cultivation by 46%, 19% and 24% (for Y_P/S_, Y_X/S_ and Y_P/X_, respectively). The space-time yield of the cultivation methods was calculated assuming a bioreactor preparation time of 24 h. For continuous cultivation, 48 h (batch operation) and the time until the steady state was reached theoretically (3 WV) were added. The runtime of the continuous cultivation was chosen so that its space-time yield equalled that of the fed-batch cultivation (25 d). Compared to batch cultivation, the space-time yield was slightly higher during fed-batch cultivation.

**Table 1:** Comparison of biomass formation and squalene production of *Schizochytrium* sp. S31 using different cultivation systems

Parameter	Batch	Fed-batch	Continuous (0.025 h^−1^)
Dry matter (g/L)	17.34	31.03	6.70 ± 0.05
Biomass productivity (g/L*h^−1^)	0.200	0.145	0.175 ± 0.001
µ (h^−1^)	0.041	0.043	0.025
Squalene content (mg/g)	29.18	38.35	39.67 ± 1.34
Squalene yield (mg/L)	559	1131	278 ± 11
Q_p_ (mg/L*h^–1^)	5.82	5.23	6.94 ± 0.27
q_p_ (mg/g*h^–1^)	0.30	0.17	1.00 ± 0.03
Y_P/S_ (mg/g_Glc_)	11.37	10.33	19.12 ± 1.10
Y_X/S_ (g/g_Glc_)	0.39	0.29	0.48 ± 0.03
Y_P/X_ (mg/g)	30.00	37.62	39.67 ± 1.34
Space-time yield (mg/L*h^–1^)	4.66	4.71	4.72 ± 0.18

Productivities (volumetric squalene productivity *Q*_*p*_ and specific squalene productivity *q*_*p*_), overall yield coefficients (squalene and biomass yield coefficients based on the substrate *Y*_*P/S*_ and *Y*_*X/S*_ and squalene yield coefficient based on the biomass *Y*_*P/X*_) and space-time yields shown for batch and fed-batch cultivation were calculated for the time point of maximal squalene yields (96 h and 216 h, respectively). Space-time yields were calculated assuming a preparation time of 24 h for batch and fed-batch cultivations. For continuous cultivation, 48 h of batch operation and the time until the steady state was reached theoretically were added. The runtime of the continuous cultivation (25 d) was chosen to equal the space-time yield reached by the fed-batch cultivation

## Discussion

### Growth monitoring by backscatter light in shake flask cultivations

In this work, the effect of oxygen supply on growth as well as squalene and sterol content/yield of *Schizochytrium* sp. S31 was studied. Offline data were supported by online backscatter and respiratory data. The investigated thraustochytrid strain is known to form cell clusters, especially at higher cell densities (Ou et al. 2023). This behaviour leads to inaccurate growth determination via optical density measurements. Growth monitoring by backscatter light is a relatively new technique, which provides more accurate and non-invasive data for filamentous/cluster-forming organisms than OD600 measurements (Bauer et al. 2022). To the best of our knowledge, CGQ measurements have not been described for thraustochytrids yet.

The relationship between optical density/dry matter and backscatter light is strain and growth phase dependent (Latimer and Pyle 1972). More data points would be necessary for a detailed examination of the correlation in this work, but the linear connection for higher biomasses is noticeable (Fig. 2, after approximately 4 g/L). The same has been demonstrated for *S. cerevisiae* and *A. fumigatus* (Bauer et al. 2022; Bruder et al. 2016). The last datapoint for each data set was excluded from the graph because the backscatter signal decreased slowly after the stationary phase was reached. This was most likely caused by a change in cell morphology and/or evaporation of the cultivation medium. It also has to be noted that the backscatter signal always depends on the flask filling volume. Therefore, a direct comparison of values between samples with different filling volumes is not possible. The course of the curve has to be evaluated experimentally.

Compared to the gravimetrically determined dry matter, the online backscatter measurements enabled considerably more detailed monitoring of the organism’s growth phases. The shift to the stationary phase took place shortly after the depletion of glucose. The exact moment of the start of the stationary phase is rarely observed by offline monitoring. Gravimetrically determined biomasses and measurements of backscatter light complement each other and enable a more in-depth analysis of culture growth.

### Monitoring of metabolic activity by respiratory data in shake flask cultivations

Online respiratory data (OTR, CTR and RQ) were obtained by off-gas analysis through a KuhnerTOM system connected to the shake flasks. Online measurements of respiratory data in shake flasks have not been described for thraustochytrids yet. However, respiratory data were used to characterise bioreactor cultivations for DHA production in *Schizochytrium* sp. (Chang et al. 2014; Guo et al. 2016, 2020). In our work, the OTR was measured in shake flasks and the OUR in the bioreactor. Both values are comparable under oxygen-limited conditions: For sufficient oxygen supply, the OTR in the cultivation vessel must be equal to or higher than the OUR. OUR ≈ OTR can be assumed for oxygen-limiting conditions because OUR is restricted by OTR (Garcia-Ochoa et al. 2010).

Optical sensors enable the measurement of dissolved oxygen (DO) in shake flasks. However, DO drops to approximately 0% under oxygen-limiting conditions, no matter how severe the limitation is. In contrast, the oxygen concentration transferred to the liquid phase (OTR) or to the inside of the cells (OUR) per time interval can still be quantified, even if the concentration of dissolved oxygen in the medium is very low. In this work, oxygen-limited conditions were observed in all shake flask cultivations of *Schizochytrium* sp. S31 as indicated by the restricted OTR curves (Fig. 1C). Slightly rising OTR courses (between 14 h and the stationary phase) in the 40-mL and 60-mL cultivations could be contributed to the release of metabolites, which changed the oxygen capacity of the cultivation medium. Additionally, a shift in OTR during this period could have been caused by decreasing filling volumes (maximal decrease of 5 mL after 250 h) due to the prolonged cultivation, which affects samples more the lower their volume is. The differences in oxygen limitation severity, however, are clearly discernible from the different heights and lengths of the OTR plateaus. RQ, as the ratio of CER/CTR and OUR/OTR, provides further information about the metabolic activity of the cultivated organism. Guo *et al*. described a positive correlation between RQ and the main product, docosahexaenoic acid (DHA) (Guo et al. 2016, 2020). This means that the RQ can be used as an indicator of product quantities for bioprocess optimization. In contrast to DHA, no general correlation between RQ and squalene accumulation was observed. In our work, a positive correlation between squalene yield and RQ was found. However, this was not confirmed by Guo *et al*. (squalene content determined as proportion of total lipids).

### Influence of oxygen supply on squalene and sterol formation in shake flasks

In this study, the maximum squalene content and yield were achieved in shake flask cultivations with the lowest oxygen supply and *vice versa* for sterol content and yield (Fig. 1 and Supplementary Fig. S1). These findings were also described for thraustochytrid ACEM 6063 by Lewis et al. (2001), albeit without relation to growth phases or respiratory data. Lack of oxygen likely reduces the activity of the oxygen-dependent squalene monooxygenase, which leads to the accumulation of the intermediate squalene and a decrease in sterol production. However, the drop in squalene content and yield during the stationary phase cannot be explained by an increase in sterol biosynthesis (Fig. 1E and Supplementary Fig. S1B). The decrease was also observed by Zhang et al. (2022) and could be due to squalene consumption as a carbon source or conversion into other products. Other microorganisms like bacteria (Rontani et al. 2003; Seo et al. 1981) and yeast (Bhattacharjee et al. 2001) are known to use squalene as a carbon source. Figure 1D shows that the RQ did not drop to its starting value at the beginning of the stationary phase, but rather to about 0.5, indicating remaining respiratory activity. This suggests that *Schizochytrium* sp. S31 was able to keep up maintenance metabolism after the exhaustion of the main carbon source glucose. Therefore, squalene or other lipids could have been used as an alternative carbon source during the stationary phase, as an RQ of below 1 indicates the catabolism of reduced substrates (Heyman et al. 2020).

### Influence of oxygen limitation on squalene formation in different bioreactor cultivation systems

**Fig. 4:**
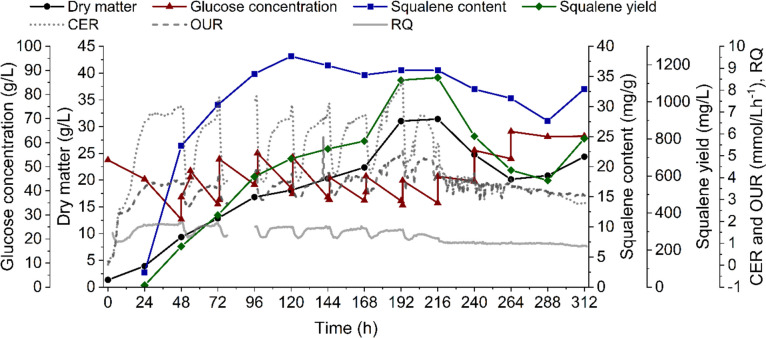
Fed-batch cultivation of *Schizochytrium* sp. S31 in a stirred tank reactor under oxygen-limited conditions. Glucose and yeast extract were fed every 24 h after an initial starting phase of 48 h. Depicted is the dry matter (black circles), glucose concentration (red triangles), squalene content (blue squares), squalene yield (green diamonds), carbon dioxide evolution rate (CER, dotted line), oxygen uptake rate (OUR, dashed line) and respiratory quotient (RQ, solid line) over cultivation time. Between 78 and 96 h, data recording stopped due to an error. Bioreactor functionality was not affected

A low oxygen supply was proven effective for boosting squalene accumulation in *Schizochytrium* sp. S31, oxygen-limiting conditions were transferred to the bioreactor scale. The oxygen supply was similar to that of the 60-mL shake flask cultivations as OTR (shake flaks) and OUR (bioreactor) were comparable (4 mmol/L*h^−1^ and 4.5 mmol/L*h^−1^, respectively). The dry matter formation in both cultivation vessels was similar during batch operation (Figs. 3 and 4). The squalene content/yield, on the other hand, increased at least eightfold in the bioreactor in comparison to the shake flasks. Considerably different mass transfer and liquid mixing in the bioreactor could have been beneficial for squalene accumulation (Büchs 2001; Marques et al. 2010). The cluster-forming organism could have been separated into single cells or smaller clusters more efficiently due to higher shear forces in the bioreactor, which would improve nutrient supply.

The fed-batch bioreactor cultivation (Fig. 4) led to an increase of squalene content by 31 and a yield of 102% compared to the batch cultivation. As glucose was steadily supplemented, no utilisation of squalene as a carbon source occurred, which most likely prevented the decrease of squalene content and yield in the stationary phase that was observed in the batch setup and the shake flasks. Optimised feeding strategies can lead to even higher biomasses and squalene productivities compared to this study (Ha et al. 2017; Hoang et al. 2018). As the optimization of the feeding strategy was not the focus of this work, this will not be discussed further. Respiratory data were comparable to those of the batch cultivation. The periodic drops in RQ, OUR and CER likely indicate a sudden increase in cell growth caused by the discontinuous feeding of yeast extract, which was also described for *Mortierella alpina* by Li et al. (2018)*.*

**Fig. 5:**
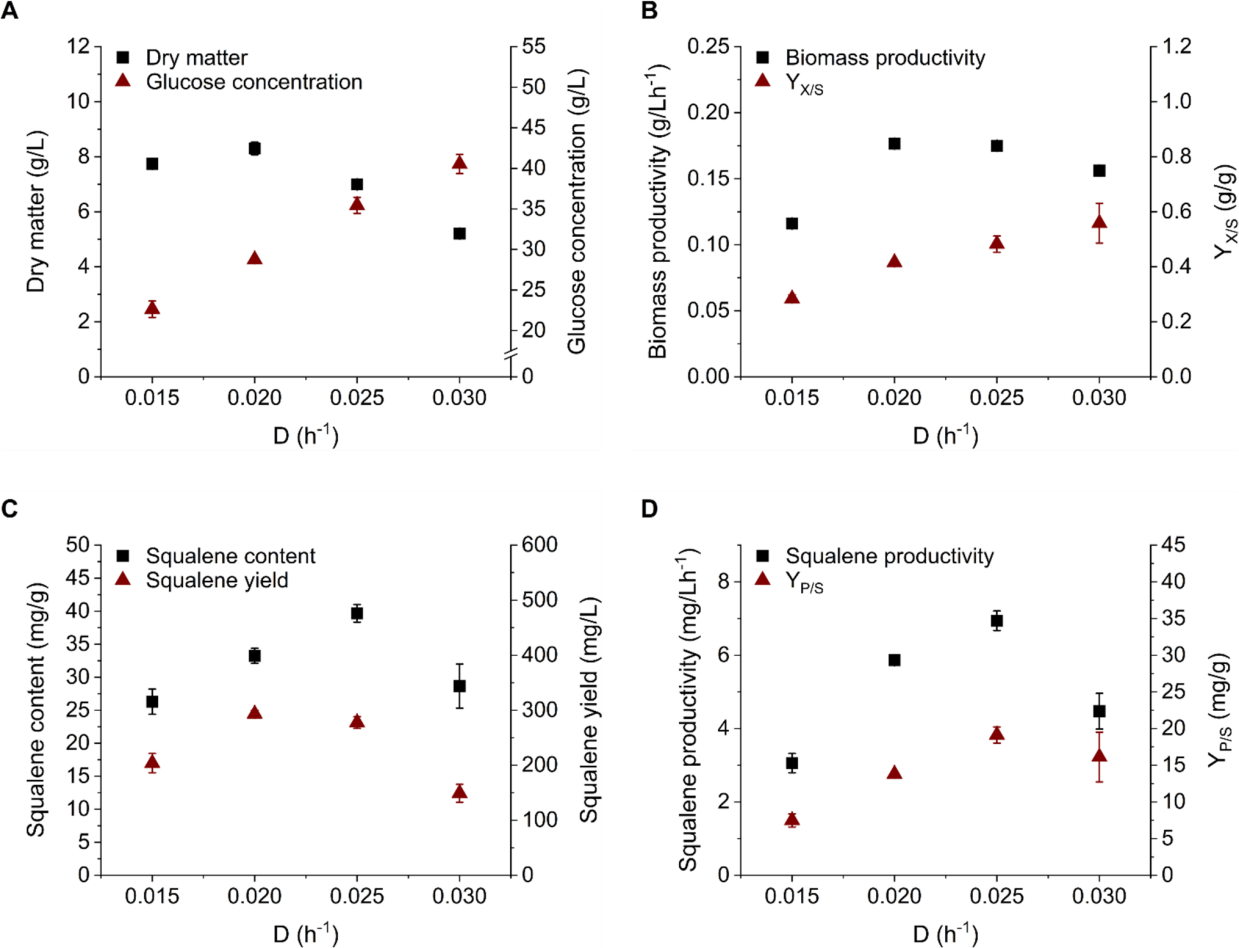
Continuous cultivation of *Schizochytrium* sp. S31 at different dilution rates. (**A**) Dry matter (black squares) and glucose concentration (red triangles), (**B**) biomass productivity (black squares) and biomass yield coefficient (red triangles), (**C**) squalene content per gramme dry matter (black squares) and squalene yield per litre culture volume (red triangles) and (**D**) squalene productivity (black squares) and squalene yield coefficient (red triangles) over the dilution rate. Data are the mean ± standard deviation of three samples taken during steady state

Continuous cultivations of thraustochytrids have not yet been described for squalene production, especially not under oxygen-limited conditions. The continuous cultivation performed in this study cannot be classified as a chemostat, as the limiting substrate (oxygen) was just a negligible part of the feed medium. Therefore, the glucose concentration increased and dry matter decreased linearly with the dilution rate due to the shortened residence time (Fig. 5). An exception to the linear behaviour of the dry matter was observed at D = 0.015 h^−1^, which can be explained by changes in lipid distribution due to sinking nutrient levels (Hoang et al. 2018; Jiang et al. 2020). Due to the opposed trend of glucose concentration and dry matter, the maximum biomass productivity was achieved at D = 0.02 and 0.025 h^−1^ and the best biomass yield coefficient at D = 0.03 h^−1^. Squalene content, squalene productivity and yield coefficient benefited the most from a dilution rate of 0.025 h^−1^, which was further used for the comparison of the cultivation systems. Because it is influenced by dry matter formation, squalene yield was somewhat higher at D = 0.02 h^−1^. In general, varying squalene accumulation among the dilution rates may be caused by different levels of certain nutrients. The type and level of nitrogen and carbon source as well as osmotic pressure influence the squalene content in thraustochytrids (Chen et al. 2010; Hu et al. 2015; Nakazawa et al. 2012).

For an overall comparison of the three cultivation systems (batch, fed-batch and continuous cultivation at D = 0.025 h^−1^), biomass and squalene productivities as well as yield coefficients were calculated (Table 1). Due to the low oxygen supply, specific growth rates were limited or defined by the dilution rate. Consequently, the growth rate of *Schizochytrium* sp. S31 could not be accelerated by feeding, which led to prolonged process times for each cultivation system. Therefore, high dry matter and squalene yields (31.03 g/L and 1131 mg/L) but low productivities in fed-batch cultivation can be explained by the elongation of cultivation time due to oxygen limitation. In general, the best biomass and squalene yield coefficients were obtained by continuous cultivation (D = 0.025 h^−1^). Low oxygen supply, sufficient nutrient levels as well as constant growth in continuous cultivation seem to be highly advantageous for squalene accumulation in *Schizochytrium* sp. S31. Space-time yields take the preparation and runtime of the system into account. The preparation time of continuous cultivation is longer compared to discontinuous cultivation because the time necessary to reach a steady state must be added. Therefore, the continuous cultivation would outperform the space-time yield of the fed-batch cultivation after 25 days.

As severe oxygen limitation had a negative effect on biomass formation and increased the runtime of the process in general, metabolic engineering could be applied to modify the sterol biosynthesis pathway, circumventing those challenges (Rau et al. 2022). Fed-batch cultivations usually outperform batch cultivations for squalene productivity. Nevertheless, under oxygen-limiting conditions, the shorter runtime of batch cultivation and the constant high squalene content during continuous cultivation could be advantageous.

## Supplementary information

Below is the link to the electronic supplementary material.

Supplementary file1 (PDF 115 KB)

## Data Availability

The datasets generated during and/or analysed during the current study are available from the corresponding author upon reasonable request.
