# Supplementary material for: Squalene production under oxygen limitation by Schizochytrium sp. S31 in different cultivation systems
Source: Appl Microbiol Biotechnol. 2024 Feb 13;108:201. doi: 10.1007/s00253-024-13051-3 (PMC10864429; doi:10.1007/s00253-024-13051-3)
Supplement: Supplementary file 1 [file 253_2024_13051_MOESM1_ESM.pdf]

Supplementary Information (SI)

Journal: Applied Microbiology and Biotechnology

Squalene production under oxygen limitation by *Schizochytrium* sp. S31 in different cultivation systems

Lina Schütte[1*], Patrick G. Hanisch[2], Nina Scheler[1], Katharina C. Haböck[1], Robert Huber[2], Franziska Ersoy[1], Ralf G. Berger[1]

[1] Institute of Food Chemistry, Gottfried Wilhelm Leibniz University Hannover, Hannover, Germany.
[2] Department of Engineering and Management, Munich University of Applied Sciences HM, Munich, Germany.
[*]Correspondence: lina.schuette@lci.uni-hannover.de

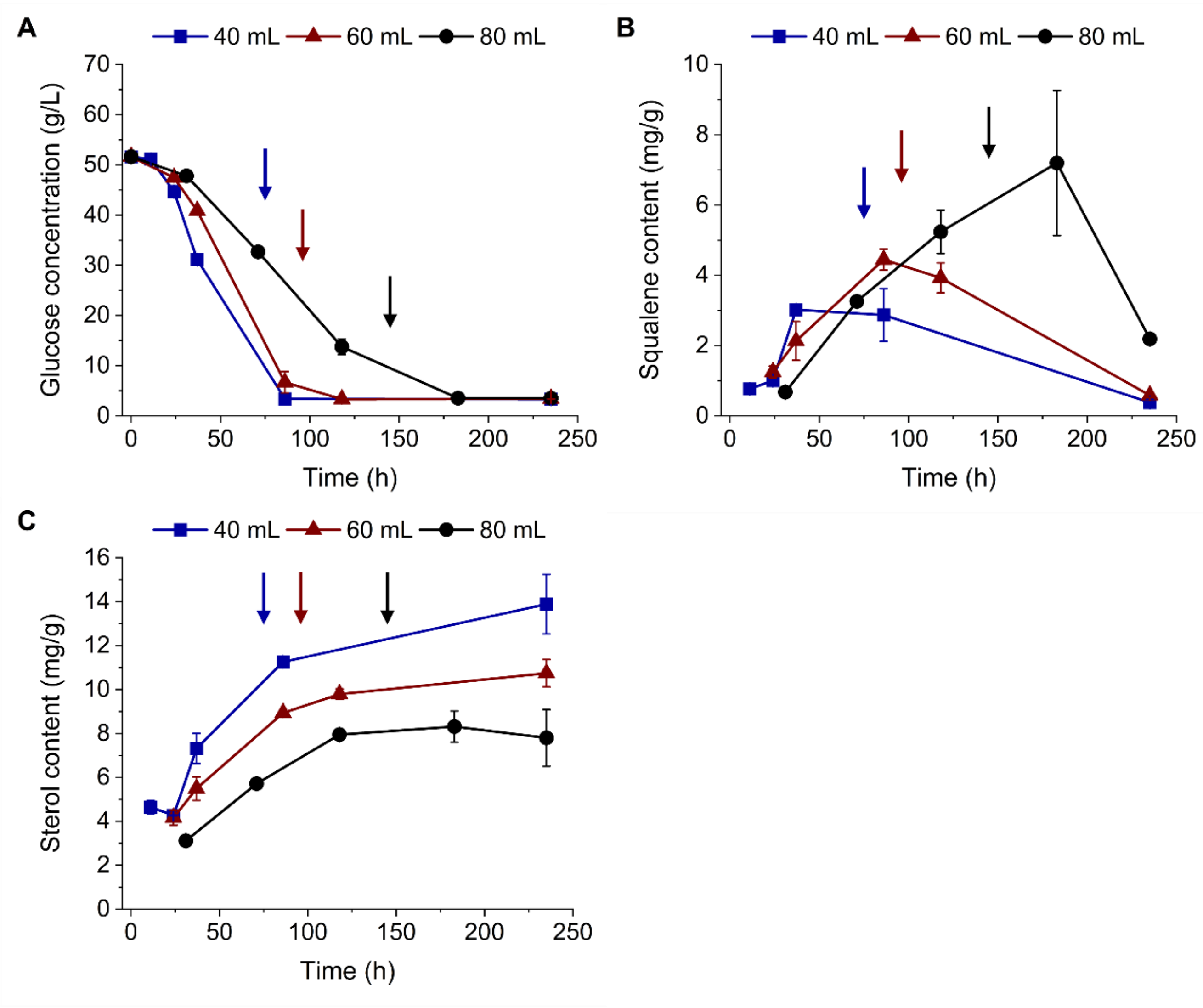

Fig. S1 Effect of different oxygen transfer rates on biomass formation and squalene/sterol accumulation of *Schizochytrium sp.* S31 in shake flasks with varying filling volumes (blue: 40 mL; red: 60 mL; black: 80 mL). Arrows indicate the start of the stationary phase for each filling volume. (**A**) Glucose concentration, (**B**) squalene and (**C**) sterol content per gramme dry matter over cultivation time**.** Data presented are the mean of duplicates ± standard deviation cultivations
